# Effects of dopamine D_4 _receptor antagonist on spontaneous alternation in rats

**DOI:** 10.1186/1744-9081-4-49

**Published:** 2008-10-22

**Authors:** Anette Moustgaard, Jann Hau, Nanna M Lind

**Affiliations:** 1 Department of Experimental Medicine, University of Copenhagen and University Hospital of Copenhagen, Denmark

## Abstract

**Background:**

The present study was a component of a series of studies scrutinising the neuroreceptor substrate of behavioural flexibility in a rat model. Spontaneous alternation paradigms model the natural tendency of rodents to spontaneously and flexibly shift between alternative spatial responses. In the study it was tested for the first time if the neurochemical substrate mediating spontaneous alternation behaviour includes the dopamine D_4 _receptor.

**Methods:**

The acute effects of the highly selective dopamine D_4 _receptor antagonist L-745,870 on rats' performance in a spontaneous alternation paradigm in a T-maze were examined. The paradigm was a food-rewarded continuous trial procedure performed for 20 trials.

**Results:**

The spontaneous alternation rate was not affected by the doses of the drug administered (0.02 mg/kg; 0.2 mg/kg; 2 mg/kg), but the position bias of the group receiving the highest L-745,870 dose (2 mg/kg) was significantly increased compared to the group that received the lowest dose (0.02 mg/kg). No significant effects on position bias were found compared to saline. The drug did not increase response perseveration.

**Conclusion:**

The results show that the neural substrate mediating the spatial distribution of responses in the spontaneous alternation paradigm includes the D_4 _receptor. However, the statistically significant effect of L-745,870 on position bias was found comparing a high drug dose with a low drug dose, and not comparing the drug doses with saline. For the tested doses of L-745,870 the effect on position bias was not large enough to affect the alternation rate.

## Background

In several neuropsychiatric disorders, e.g. schizophrenia and obsessive compulsive disorder, the neuropsychological symptoms include impaired performance on tasks requiring behavioural shifting [1,2], pointing to deficits in cognitive or behavioural flexibility. In order to develop drugs that can stimulate behavioural flexibility in such brain disorders, more knowledge about the neurochemical basis of cognitive and behavioural flexibility in the healthy brain is called for [e.g. [3]]. The present study was a component of a series of studies scrutinising the neuroreceptor substrate of behavioural flexibility in a rat model.

Spontaneous alternation paradigms model the natural tendency of rodents to spontaneously and flexibly shift between alternative spatial responses. We hypothesize, that spontaneous behavioural flexibility may rely on different neural mechanisms than the behavioural flexibility required for response shifting in learned tasks. It is therefore relevant to study the neural basis of spontaneous alternation in addition to studying the neural mechanisms involved in various learned tasks requiring behavioural flexibility.

The dopamine D_4 _receptor is expressed in relatively high amounts in the cerebral cortex as well as in structures such as hippocampus, amygdala and hypothalamus. In the neocortex, it is especially abundant in the prefrontal cortex of both monkeys and rats [4,5]. Behavioural flexibility – especially the ability to shift and inhibit responses and behavioural strategies – has been associated with the prefrontal cortex [6,7] and recently also with the D_4 _receptor [8]. In studies of spontaneous alternation, prefrontal lesions [9] and administration of prefrontally active non-selective dopaminergic drugs such as *d*-amphetamine [10,11] reduced the alternation rate, and in some cases also increased position bias [12]. Both of these effects may reflect reduced behavioural flexibility. Thus, there is evidence for a role of prefrontal and dopaminergic neural mechanisms in the mediation of spontaneous alternation behaviour, but several other neurotransmitter systems and neuroanatomical structures play important roles as well. Cholinergic antagonists – especially M1 receptor antagonists – and lesions of structures with considerable cholinergic innervation such as the hippocampus, reduces alternation rate. But also the NMDA receptor, serotonergic receptors and various neuropeptide systems are involved in the mediation of spontaneous alternation behaviour [11].

Infusion of the highly selective D_4 _receptor antagonist L-745,870 [13] into the medial prefrontal cortex of the rat has recently been reported to improve set shifting ability, pointing to a role of prefrontal D_4 _receptors in mediating aspects of behavioural flexibility [8]. L-745,870 has also been found to have an effect on working memory in a delayed alternation paradigm, possibly via its effect on the prefrontal cortex [14]. The effect of D_4 _antagonists on spontaneous alternation has not been studied hitherto. In the present study we examined the acute effect of L-745,870 on spontaneous alternation in rats.

We believed that it was most likely that L-745,870 would decrease behavioural flexibility through disturbance of dopaminergic processes in the rat prefrontal cortex. We therefore hypothesised, that administration of L-745,870 would decrease alternation rate and increase position bias in the spontaneous alternation paradigm as has previously been found after administration of non-selective dopaminergic drugs [12].

## Methods

### Subjects

A total of 32 male Wistar rats (Charles River Labs., Sulzfeld, Germany), weighing approximately 250 g at the start of the experiment, were used. Female rats were not included in the study to avoid confounding variables associated with oestrus cycle variation. The rats were housed singly in Macrolone Type III cages (Scanbur, Køge, Denmark) with aspen bedding (Tapvei Estonia, Harjumaa, Estonia) in a temperature (21°C +/- 1°C) and humidity (45–65%)-controlled environment and maintained at a 12 h/12 h light/dark cycle. Testing was conducted during the light phase. Daily food intake of Altromin 1314 (Altromin GmbH, Lage, Germany) was restricted, and the rats weighed 90% of their free-feeding weight on the day of drug testing. Acidified (citric acid) tap water was available ad libitum. The experiments were approved by the Danish Ministry of Justice and were in compliance with the European Communities Council directive 86/609/EEC and the recently revised Appendix A to the Council of Europe Convention ETS 123.

### Apparatus

The apparatus was a beige-coloured Plexiglas T-maze (width 10 cm; height 20.5 cm; length of start alley 38 cm; length of response arms 30.5 cm) without start box. Three cm into each of the response arms a vertical flap was placed covering the width of the arm. The rats had to run through the flap in order to gain access to food. Each flap was made of plastic coated white paper and suspended from a horizontal string. The maze was placed in a dimly lit room without ceiling lights. Light sources were placed symmetrically around the maze.

### Habituation

Habituation started on the second day of the food deprivation period. All rats were subjected to one 20 min habituation session in the T-maze on each of three consecutive days. During habituation, the rats were allowed to explore the maze freely with mashed food (1/3 rat chow, 2/3 water) present at the end of both response arms. The experimenter was located at the end of the start alley. The rats were weighed before and after the sessions to determine their food intake in the maze. After each habituation session all rats were subjected to a 5 min handling session.

### Drug treatment

The rats were randomly assigned to four groups injected i.p. with either 0 μg/kg, 20 μg/kg, 200 μg/kg, or 2000 μg/kg 3- [4-(4-chlorophenyl)piperazin-1-yl-1H-pyrrolo[2,3-b]pyridine hydrochloride (L-745,870) (Sigma-Aldrich, Germany) diluted in isotonic saline. In accordance with [14], the rats were injected 40 min prior to testing. Injection volumes were 10 ml/kg body weight. In the post injection pause the rats were placed in their home cages in a rodent container (Scantainer, Scanbur, Køge, Denmark) in the testing room. The drug doses were randomised with respect to the testing day and time of the day, according to a between-subject Latin square design. However, one rat was by a mistake injected with 20 μg/kg instead of 2000 μg/kg L-745,870. Thus, the group sizes were as follows: 0 μg/kg (n = 8), 20 μg/kg (n = 9), 200 μg/kg (n = 8), 2000 μg/kg (n = 7). The experimenter was always kept ignorant regarding the treatment given to the individual animals.

### Drug testing

On the drug testing day the rats were given 20 trials of running in the T-maze. The rats could freely choose between the two response arms of the maze. On each trial the rat was released into the start alley of the maze and was allowed to run through one of the flaps into a response arm. After an eating period of approximately 8 s, the rat was picked up and placed in a small transport cage next to the maze. If a rat ran back from a response arm through a flap, the rat was picked up, whether it had eaten or not. The rats were not allowed to run into two response arms during the same trial. Inter-trial intervals were approximately 15 s including the eating period. If a rat could not complete 20 trials in 60 min, its data were discarded from the experiment (this happened with one rat in the 20 μg/kg group).

### Data and data analysis

For each rat, raw data consisted of the sequence of the 20 consecutive right or left responses in the T-maze. A response counted as soon as a rat ran from the start alley through one of the flaps of the response arms, regardless of whether it subsequently ate or not. Very rarely the experimenter could not prevent a rat from running into two different maze arms during the same trial. In these coincidences, the two arm visits counted as if they were responses of two consecutive trials. The percentage alternation (percentage of responses in which the rat shifted side compared to the last response) and the percentage position bias (percentage of responding to the side of the maze preferred by that individual rat) were calculated for each rat for each of the following sections of the session: all 20 trials, the first six trials (first five possible alternations), the first 10 trials, the last 10 trials, and the last six trials. The first six trials were analysed separately as the alternation rate is highest for these initial trials. In order to test for perseveration tendencies, two additional parameters were considered for each rat (for all 20 trials): (1) the maximum number of consecutive responses to the same side of the maze, and (2) the number of response sequences consisting of at least three consecutive responses to the same side of the maze. The hypotheses were that L-745,870 would decrease alternation rate, increase position bias, and increase response perseveration. Results of the four dosage groups were compared using one-way between-subject ANOVA (*p *= .05 significance level), and only if significant differences were revealed, the groups were compared in pairs using post hoc Tukey's multiple comparisons test (*p *= .05 significance level).

## Results

**Figure 1:**
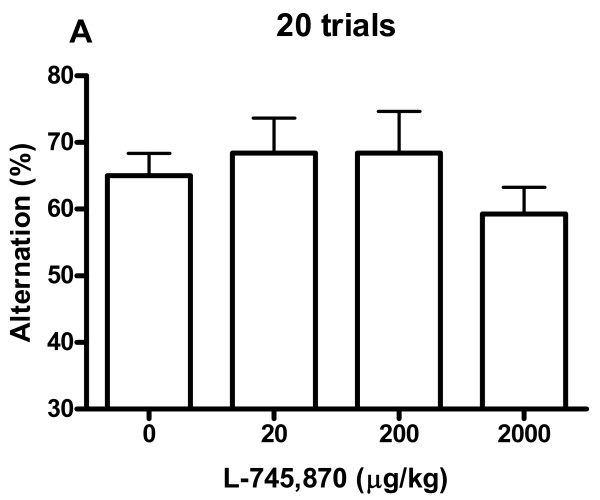
**The percentage spatial alternation after different doses of L-745,870**. All 20 trials included. Data represent mean ± S.E.M. No significant differences.

**Figure 2:**
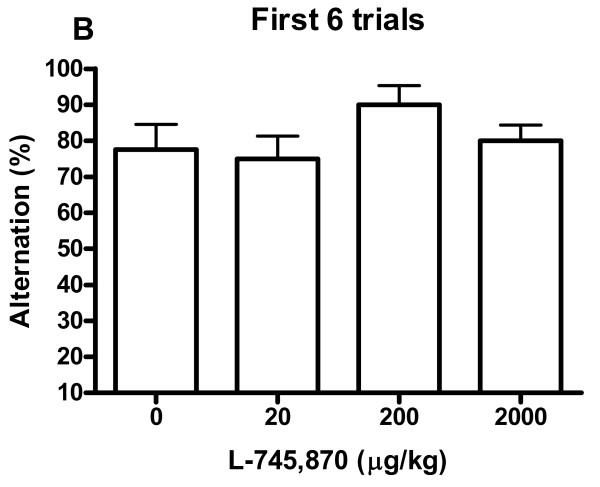
**The percentage spatial alternation after different doses of L-745,870**. First six trials included. Data represent mean ± S.E.M. No significant differences.

**Figure 3:**
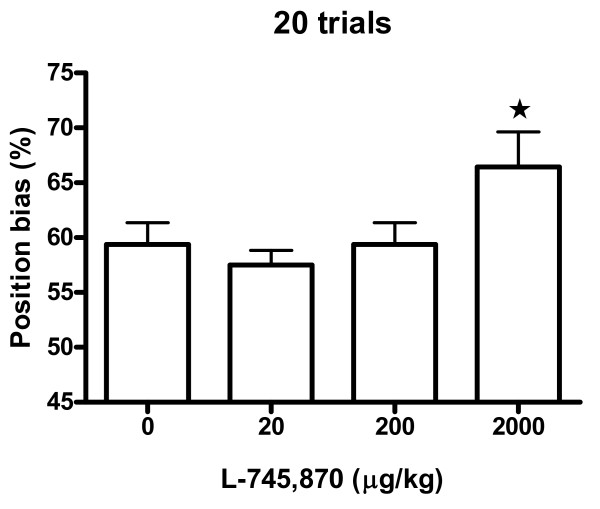
**The percentage position bias after different doses of L-745,870**. All 20 trials included. Data represent mean ± S.E.M. *: significantly different from the 20 μg/kg group (*p *< .05).

Spatial alternation rates are shown in Fig. 1 and Fig. 2. For the saline group there was a mean alteration rate of 78% for the first six trials, and 65% for all 20 trials. No differences between the four dosage groups were revealed by ANOVA for alternation rate, neither for the entire session (20 trials, Fig. 1, *p *= .55, *F*(3,27) = 0.72), nor for any of the considered session parts (first six trials, Fig. 2: *p *= .30, *F*(3,27) = 1.27; first 10 trials: *p *= .74, *F*(3,27) = 0.42; last 10 trial: *p *= .69, *F*(3,27) = 0.50; last 6 trials: *p *= .33, *F*(3,27) = 1.19). Significant differences in position bias were found between the four dosage groups when analysing all 20 trials (*p *< .05, ANOVA, *F*(3,27) = 3.11) (Fig. 3). Comparisons of groups in pairs showed that the position bias of the 2000 μg/kg group was significantly increased compared to the 20 μg/kg group (*p *< .05, post hoc Tukey's multiple comparisons test). For the other considered session parts no differences in position bias were revealed by ANOVA (first 6 trials: *p *= .38, *F*(3,27) = 1.08; first 10 trials: *p *= .07, *F*(3,27) = 2.61; last 10 trials: *p *= .78, *F*(3,27) = 0.36; last 6 trials: *p *= .48, *F*(3,27) = 0.86). Of the rats with a position bias of at least 60% (in any dosage group), 65% preferred the right arm. ANOVA did not reveal any differences between the dosage groups for any of the perseveration parameters (maximum number of consecutive responses to the same side of the maze: *p *= .87, *F*(3,27) = 0.24; number of response sequences consisting of at least three consecutive responses to the same side of the maze: *p *= .71, *F*(3,27) = 0.46). One rat in the 20 μg/kg group could not complete 20 trials in 60 min and its data were discarded from the experiment. This rat already during the habituation sessions exhibited poor habituation to the maze and ate very sparsely.

## Discussion

The highest dose of the D_4 _antagonist L-745,870 (2000 μg/kg) moderately, but statistically significantly, increased position bias in the present spontaneous alternation paradigm compared to the lowest dose (20 μg/kg) (Fig. 3). Rats injected with 2000 μg/kg L-745,870 also had a higher spatial bias than the saline group and the 200 μg/kg group, but these differences did not reach statistical significance. Thus, the hypothesis that L-745,870 would increase position bias was confirmed by the comparison of high and low L-745,870 doses, but not by the comparison of L-745,870 and saline. It was also the hypothesis, that the D_4 _antagonist would decrease the spatial alternation rate. There were, however, no significant differences in the alternation rate between the dosage groups for any parts of the session. Thus, the latter hypothesis was not confirmed.

This combination of results can only have been achieved because the effect of the drug doses tested on position bias was moderate. Had the increasing effect on position bias been more substantial, the alternation rate would have been decreased as a logical consequence. Control rats alternated at a relatively high rate (78%) during the first six trials of the experiment, and still over chance level (65%) when all 20 trials were considered. Thus, there should be no hindrance of showing a possible decrease in alternation rate due to floor effect. Alternation rates of 60–70% are usual in spontaneous alternation paradigms, depending on the behavioural strategy used by the rats [15]. However, many versions of spontaneous alternation test regimes – regarding apparatus and procedure – have been described in the literature, some with alternation rates as high as 93% [e.g. [16]]. Therefore it cannot be excluded that it may be possible to demonstrate a decreasing effect of the D_4 _antagonist – in the doses employed in the present study – on alternation rate, using another spontaneous alternation paradigm. However, the behavioural strategy used by rats in some spontaneous alternation paradigms with very high alternation rates probably differs from the "standard" spontaneous alternation strategy. For instance, Pych et al. reported that rats exhibiting 93% alternation in a Y-maze paradigm used a persistent turning strategy [16]. Furthermore, the moderate alternation rate obtained with the present testing paradigm leaves room for increasing as well as decreasing effects of drugs on the alternation rate. An increasing effect of L-745,870 on alternation rate was a possibility in the present experiment, as the drug seemed to improve the behavioural shifting ability of rats in an earlier study [8].

We cannot exclude that the highest drug dose (2 mg/kg L-745,870) could have mildly impaired locomotor activity, and that this motor impairment could have affected behaviour in the spontaneous alternation test. We did not measure the effect of L-745,870 on locomotor activity in the present study. However, visual observation suggested no obvious locomotor suppressing effects of the drug in any of the doses used. Patel et al. reported that 1 mg/kg orally dosed L-745,870 had no effect on locomotor activity in squirrel monkeys, while 10 mg/kg greatly reduced locomotor activity, induced sedation and mild parkinsonism [13]. The motor impairing effects of L-745,870 are most likely due to the antagonist binding to D_2 _receptors, when the drug level in the brain is high [13]. At the dose range used in the present study, L-745,870 has negligible action at other than D_4 _receptors [13], and significant locomotor suppressing effects were therefore not expected in the experiment. Ideally, the highest dose of drugs used in behavioural pharmacology studies should have a noticeable effect on generalised behaviour. However, the L-745,870 doses of the present study were chosen, because the D_4 _antagonist is highly selective in these doses, and significant effects on performance of a learned alternation task have been found previously using comparable doses of L-745,870 (Zhang et al., 2004).

McFarland (1989) reported an increasing effect on position bias in a spontaneous alternation paradigm after administration of other drugs acting on the dopaminergic system, i.e. the dopamine releaser *d*-amphetamine and the non-specific dopamine receptor agonist apomorphine [10]. However, in these studies the increasing effect on position bias was followed by a decrease in alternation rate. It has been suggested, that the effects of *d-*amphetamine and dopaminergic agonists on spontaneous alternation may be due to drug-induced behavioural perseveration [10]. Since behavioural perseveration is one of the clearest manifestations of behavioural inflexibility that can be seen in behavioural experiments, we also analysed for repetitive position responding in the present study. There was, however, no increase in perseverative behaviour after administration of L-745,870 in the present study. Repetitive position responding for 5–10 trials in a row occurred in all groups – also the saline group – in a minority of the rats.

The effect of the D_4 _receptor antagonist on spontaneous alternation could also have been tested using a paradigm in which the attenuation by the D_4 _antagonist of behavioural disruptions induced by a non-specific indirect agonist such as *d-*amphetamine was investigated. Such a paradigm could be applied to – and could prove to be valuable for – future studies of the effect of selective dopamine antagonists on spontaneous alternation, since *d-*amphetamine has been demonstrated to disrupt normal behaviour in spontaneous alternation tests [12].

A broad spectrum of doses of L-745,870 comparable to the doses used in the present study has earlier been shown to have either positive or negative effect on delayed alternation performance, depending on delay lengths as well as the baseline performance of the rats [14]. The effects were interpreted as effects on working memory. Even though working memory is not the main topic of the present study, it is noteworthy that L-745,870 did not seem to interfere with working memory in the present study since the drug did not change the alternation rate. Working memory is involved in the rats' recollection of their latest response in spontaneous alternation. The inter-trial interval, however, was of shorter duration in the present study (15 s) than in the study of Zhang and colleagues [14].

The dopaminergic drugs that have earlier been found to affect spontaneous alternation [17,18] were not receptor-selective, and not much can therefore be concluded with certainty about the type of dopamine receptors involved. D_1 _receptor knockout mice have been tested in spontaneous alternation and did not differ from controls [19], but functional compensation processes during development probably play a large role in knockout animals. It seems that the administration of dopaminergic agonists e.g. quinpirole [18] and antagonists e.g. pimozide [17] as well as selective depletions of dopamine can result in decreased spontaneous alternation rate [11]. These results indicate that alternation behaviour is dependent upon an optimal balance in a distributed dopaminergic network.

In conclusion it has been demonstrated for the first time that the D_4 _receptor antagonist L-745,870 had an effect on position bias in a spontaneous alternation paradigm. It is too early to say if the effect on position bias found in the present study after administration of a relatively high dose of L-745,870 should be interpreted as a mild decrease in behavioural flexibility, related to the effects seen after prefrontal lesions or the administration of *d-*amphetamine. In future studies addressing this issue, it would be relevant to test higher doses of L-745,870 than 2 mg/kg in the present spontaneous alternation paradigm, and to test the effect of D_4 _antagonists on other spontaneous alternation paradigms, e.g. non-reinforced or discrete-trial paradigms. Paradigms using attenuation by D_4 _antagonists of dopamine agonist-induced disruption of behaviour should also be performed.

## Competing interests

The authors declare that they have no competing interests.

## Authors' contributions

AM wrote the manuscript and participated in the design of the study, data acquisition, data analysis, and data interpretation. NML helped revising the manuscript, participated in the design of the study, data acquisition, data analysis, and data interpretation. JH helped revising the manuscript and participated in data analysis and data interpretation. All authors read and approved the final manuscript.
